# Development and Validation of a Novel Prognosis Prediction Model for Patients With Stomach Adenocarcinoma

**DOI:** 10.3389/fmed.2021.793401

**Published:** 2021-12-22

**Authors:** Tong Wang, Weiwei Wen, Hongfei Liu, Jun Zhang, Xiaofeng Zhang, Yu Wang

**Affiliations:** ^1^School of Life Sciences, Zhejiang Chinese Medical University, Hangzhou, China; ^2^Department of Dermatology, Third People's Hospital of Hangzhou, Hangzhou, China; ^3^Department of Zhiweibing, Ningbo Municipal Hospital of Traditional Chinese Medicine, Ningbo, China; ^4^Department of Gastroenterology, Affiliated Hangzhou First People's Hospital, Zhejiang University School of Medicine, Hangzhou, China; ^5^Hangzhou Institute of Digestive Diseases, Hangzhou, China; ^6^Key Laboratory of Integrated Traditional Chinese and Western Medicine for Biliary and Pancreatic Diseases of Zhejiang Province, Hangzhou, China

**Keywords:** stomach adenocarcinoma, GEO, TCGA, differentially expressed genes, prognostic model

## Abstract

**Background:** Stomach adenocarcinoma (STAD) is a significant global health problem. It is urgent to identify reliable predictors and establish a potential prognostic model.

**Methods:** RNA-sequencing expression data of patients with STAD were downloaded from the Gene Expression Omnibus (GEO) and the Cancer Genome Atlas (TCGA) database. Gene expression profiling and survival analysis were performed to investigate differentially expressed genes (DEGs) with significant clinical prognosis value. Overall survival (OS) analysis and univariable and multivariable Cox regression analyses were performed to establish the prognostic model. Protein–protein interaction (PPI) network, functional enrichment analysis, and differential expression investigation were also performed to further explore the potential mechanism of the prognostic genes in STAD. Finally, nomogram establishment was undertaken by performing multivariate Cox regression analysis, and calibration plots were generated to validate the nomogram.

**Results:** A total of 229 overlapping DEGs were identified. Following Kaplan–Meier survival analysis and univariate and multivariate Cox regression analysis, 11 genes significantly associated with prognosis were screened and five of these genes, including COL10A1, MFAP2, CTHRC1, P4HA3, and FAP, were used to establish the risk model. The results showed that patients with high-risk scores have a poor prognosis, compared with those with low-risk scores (*p* = 0.0025 for the training dataset and *p* = 0.045 for the validation dataset). Subsequently, a nomogram (including TNM stage, age, gender, histologic grade, and risk score) was created. In addition, differential expression and immunohistochemistry stain of the five core genes in STAD and normal tissues were verified.

**Conclusion:** We develop a prognostic-related model based on five core genes, which may serve as an independent risk factor for survival prediction in patients with STAD.

## Background

Approximately 1.4 million people die each year worldwide from adenocarcinomas of the esophagus, stomach, colon, or rectum (1), of which stomach adenocarcinoma (STAD) has the third highest incidence and second highest for cancer-related mortality, and it remains a significant global health problem (2). In 2018, STAD was estimated to cause one million new cases and 781,000 deaths worldwide (3). Since the non-specific symptoms in early stages of the disease, STAD is typically not diagnosed until the disease has progressed to a more severe state, resulting in poor prognosis due to metastasis, intratumoral heterogeneity, chemotherapy resistance, etc. (4). This raises an urgent need for the development of reliable diagnostic, prognostic, and therapeutic molecular biomarkers of STAD.

Integrative bioinformatics analysis is one of the frontiers of biological research today and can be used to identify differential genes, screen prognostic biomarkers, and select appropriate treatment approach (5). Research on single-gene prediction is very concentrated, but it is not yet effective in prognosis. Polygenic combination has been reported to possess better predictive ability for cancer prognosis than single genes (6). For instance, Lu et al. (7) revealed that the dysregulated expression of the THBS family was closely related to STAD prognosis and tumor immunity. Additionally, Liu et al. (8) demonstrated that the SFRP family was potential targets for precision therapy and prognostic biomarkers for survival of patients with STAD. Although there are some polygene bioinformatics analysis studies, most of them focus on predicting of signatures, and there is still a lack of research on polygenic risk estimation model and predict prognosis of STAD.

**Figure 1:**
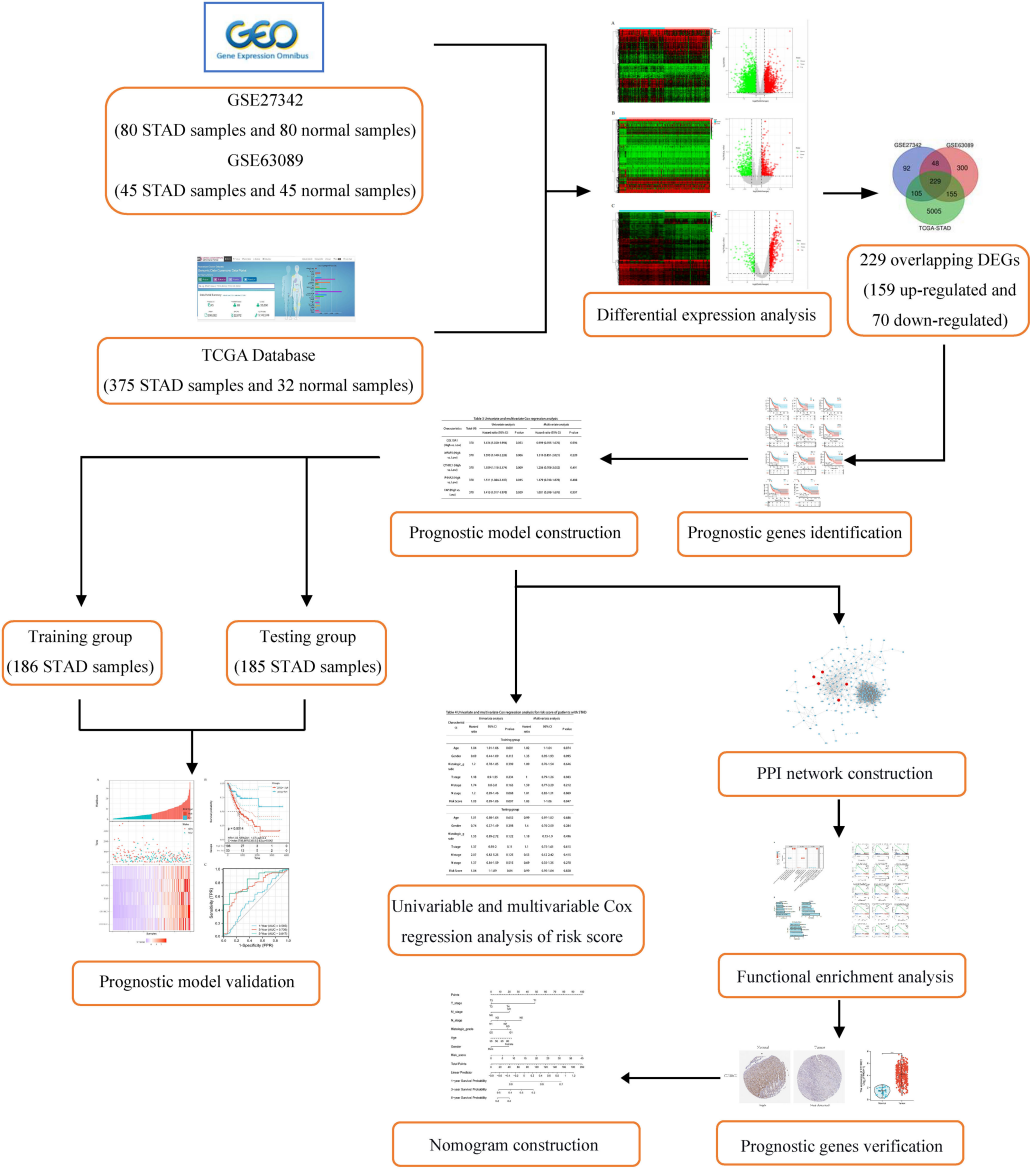
Flowchart of this study.

In this study, we aimed to develop a prognostic model for the predict prognosis for patients with STAD. A large number of mRNA expression profiles of patients with STAD were downloaded from Gene Expression Omnibus (GEO) and the Cancer Genome Atlas-Stomach Adenocarcinoma (TCGA-STAD) database. Differential expression analysis was used to identify differentially expressed genes (DEGs) between STAD-related tissue and normal tissue. Then, survival analysis and univariable Cox regression analysis were performed to screen prognostic genes, and multivariable Cox regression analysis was used to establish a prognostic risk model. Further, protein–protein interaction (PPI) network, functional enrichment analysis, differential expression, and structure investigation of the core genes were performed. Finally, a nomogram that includes age, gender, tumor TNM stage, histologic grade, and 5-gene risk prediction model as an independent clinical factor was used to predict the 1-, 3-, and 5-year survival rate of patients with STAD. The detailed flowchart of this work is provided in Figure 1.

## Methods

### Data Source

The GEO database (http://www.ncbi.nlm.nih.gov/geo) was used to retrieve data with “stomach adenocarcinoma” as the keywords and human as the species. Datasets that covered cancer tissue and normal adjacent tissue, came from the same platform, and contained at least 20 samples were selected, and then, the gene expression profiles and their clinical data were downloaded. From TCGA portal (https://tcga-data.nci.nih.gov/tcga/), we collected the STAD RNA-seq data and related clinical parameters.

### Differential Expression Analysis

Gene level expression data were normalized and then log2 transformation is provided by the limma package of R software (version 3.6.3). For GEO datasets, data were analyzed using the GEO2R analysis tool, and the DEGs were identified at adjusted *p* < 0.05 and |Log_2_FC| >1. For TCGA-STAD cohort, DEGs were identified with false discovery rate (FDR) < 0.05 and |Log_2_FC| >1 *via* the edge R package (9).

### Prognostic Genes' Identification

Overlapping DEGs were screened based on the *p*-value and fold change (FC)/log(FC), and the top 50 genes were selected for Kaplan–Meier survival analysis. Log-rank *p*-values for Kaplan–Meier plots were calculated using an R package for survival analysis. Then, we screened genes with log-rank *p* < 0.05 as prognostic-related genes for subsequent analysis.

### Construction and Validation Prognostic Model of STAD

To establish the prognostic model of STAD, univariable and multivariable Cox regression analyses were performed on the prognostic-related genes by the survival R package (10). Owing to the lack of survival information on GEO, we randomly divided the patients with complete survival information in TCGA-STAD dataset into training set and validation set, fit the model in the training set, and assessed its performance in the validation set. Then, the prognostic model was established based on corresponding coefficients of the prognostic genes of STAD.


$$\begin{array}{l} \text{Risk score}=\overset{n}{\underset{i=1}{\sum }}\beta \times \text{Ex}{\text{p}}_{i} \end{array}$$

Further, the training set was divided into high- and low-risk groups according to the median value of risk score. Kaplan–Meier survival analysis was performed to estimate overall survival (OS) between the two groups by the survival R package. Time-dependent receiver operating characteristic (ROC) curves were plotted by time ROC R package, and the areas under ROC curves (AUCs) were calculated to test the efficiency of the prognostic model (11). Univariable and multivariable Cox regression analyses were applied on clinical data (including age, gender, TMN stage, and histologic grade) and risk scores to assess whether the risk model was an independent prognostic factor of clinical parameters.

### PPI Network Construction and Functional Enrichment Analysis

Interaction network analysis was obtained by employing STRING v11.5 database (http://string-db.org/), keeping default parameters. The topological properties of the PPI network included average shortest path length, betweenness centrality, closeness centrality, degree, eccentricity, neighborhood connectivity, radiality, stress, and topological coefficient. Molecular complex detection (MCODE) analysis was applied to the prognostic-related gene network to identify densely connected subnetwork modules. Gene ontology (GO) and Kyoto Encyclopedia of Genes and Genomes (KEGG) enrichment analysis were performed to identify significant pathways *via* the “cluster Profiler” package in R (12). The items of biological processes were further analyzed by GO classifications. The adjusted *p* < 0.05 was considered to indicate a statistically significant difference. In addition, gene set enrichment analysis (GSEA) was utilized to determine the core gene-related signaling pathways by the “cluster Profiler” package in R. Results with absolute value of normalized enrichment score > 1, FDR < 0.25, and adjust *p* < 0.05 were considered statistically significant. 1D linear domain structures and 3D structures of proteins were visualized using cBioPortal (http://www.cbioportal.org/).

### Prognostic Gene Expression Investigation in STAD and Nomogram Construction

Differential expression of the prognostic genes between normal and STAD-related tissues was verified. Additionally, immunohistochemistry staining of the prognostic genes in STAD and normal tissues was acquired from the Human Protein Atlas database (https://www.proteinatlas.org/). According to the results of univariate and multivariate Cox regression analyses, a nomogram was created using the rms and survival package of R (13). Additionally, a calibration plots were generated to validate the nomogram.

## Results

### Differential Expression Analysis

**Figure 2:**
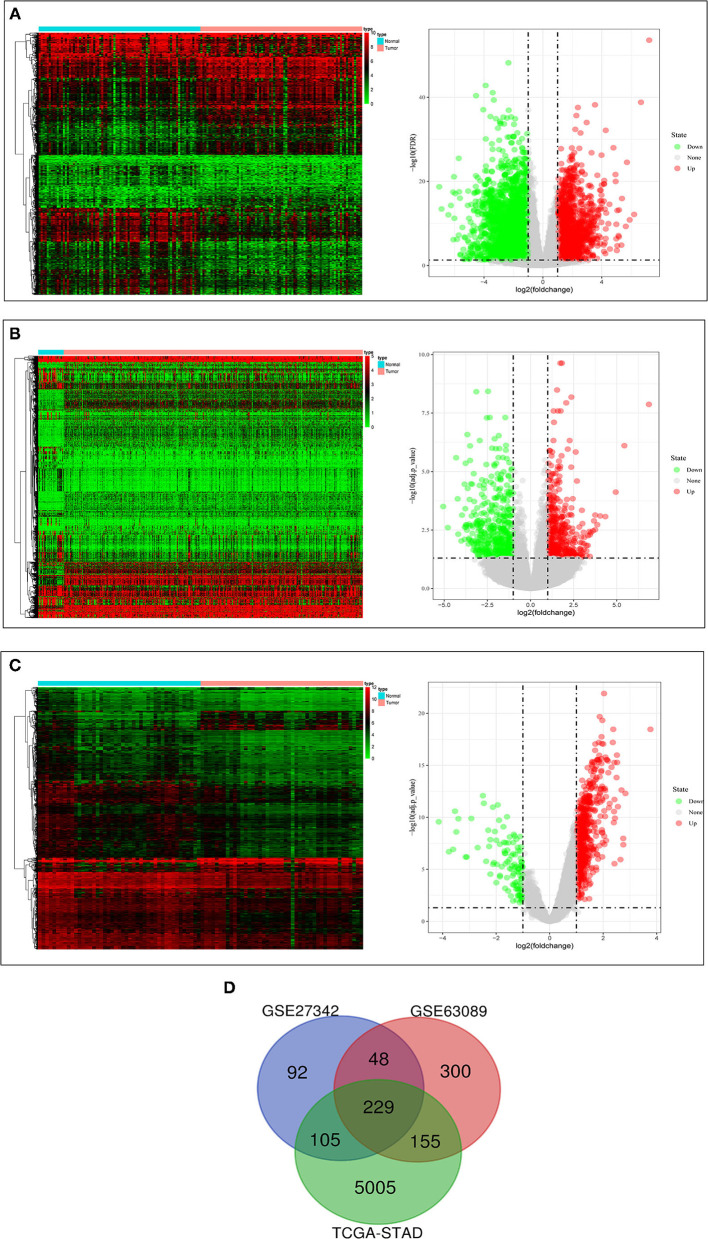
The results of differential expression analysis. **(A)** The heatmap and volcano plots visualizing the DEGs in TCGA-STAD. **(B)** The heatmap and volcano plots visualizing the DEGs in GSE27342. **(C)** The heatmap and volcano plots visualizing the DEGs in GSE63089. **(D)** Venn diagram showing the overlapping DEGs in the three datasets.

The clinical data of GSE27342, GSE63089, and TCGA-STAD were shown in Table 1. We found 474 DEGs in GSE27342 profile (287 upregulated and 187 downregulated, Figure 2A), 732 DEGs in GSE63089 profile (622 upregulated and 110 downregulated, Figure 2B), and 5,494 DEGs in TCGA-STAD cohort (2,659 upregulated and 2,835 downregulated, Figure 2C). Subsequently, a total of 229 overlapping DEGs (159 upregulated and 70 downregulated) were screened among the three datasets (Figure 2D and Additional File 1).

**Table 1:** Clinical or characteristics of patients with STAD in different datasets.

**Characteristic**	**TCGA data (*n*, %)**	**GSE27342** **(*n*, %)**	**GSE63089** **(*n*, %)**
Platform	Illumina HiSeq2000 RNA sequencing platform	Affymetrix Human Exon 1.0 ST Array	Affymetrix Human Exon 1.0 ST Array
**Samples**	**407 (100.0%)**	**160 (100.0%)**	**90 (100.0%)**
Normal	32 (7.9%)	80 (50.0%)	45 (50.0%)
Tumor	375 (92.1%)	80 (50.0%)	45 (50.0%)
**Survival status**	**377 (92.6%)**	NA	NA
Death	145 (35.6%)	NA	NA
Survival	232 (57.0%)	NA	NA
**Age**	**366 (89.9%)**	**77 (48.1%)**	**70 (77.8%)**
<=65	155 (38.1%)	59 (36.9%)	51 (56.7%)
>65	211 (51.8%)	18 (11.3%)	19 (21.1%)
**Gender**	**380 (93.3%)**	**80 (50.0%)**	**70 (77.8%)**
Female	137 (33.7%)	27 (16.9%)	25 (27.8%)
Male	243 (59.7%)	53 (33.1%)	45 (50.0%)
**Stage**	**356 (87.5%)**	**80 (50.0%)**	NA
I	55 (13.5%)	4 (2.5%)	NA
II	112 (27.5%)	7 (4.4%)	NA
III	150 (36.9%)	54 (33.8%)	NA
IV	39 (9.6%)	15 (9.4%)	NA
**T classification**	**372 (91.4%)**	NA	**70 (77.8%)**
T1	20 (4.9%)	NA	12 (13.3%)
T2	84 (20.6%)	NA	16 (17.8%)
T3	168 (41.3%)	NA	25 (27.8%)
T4	100 (24.6%)	NA	17 (18.9%)
**N classification**	**362 (88.9%)**	NA	NA
N0	113 (27.8%)	NA	NA
N1	99 (24.3%)	NA	NA
N2	76 (18.7%)	NA	NA
N3	74 (18.2%)	NA	NA
**M classification**	**358 (88.0%)**	NA	NA
M0	332 (81.6%)	NA	NA
M1	26 (6.4%)	NA	NA

*TCGA, The Cancer Genome Atlas; NA, not available; T, primary tumor; N, regional lymph nodes; M, distant metastasis*.

### Prognostic-Related Genes' Identification

**Figure 3:**
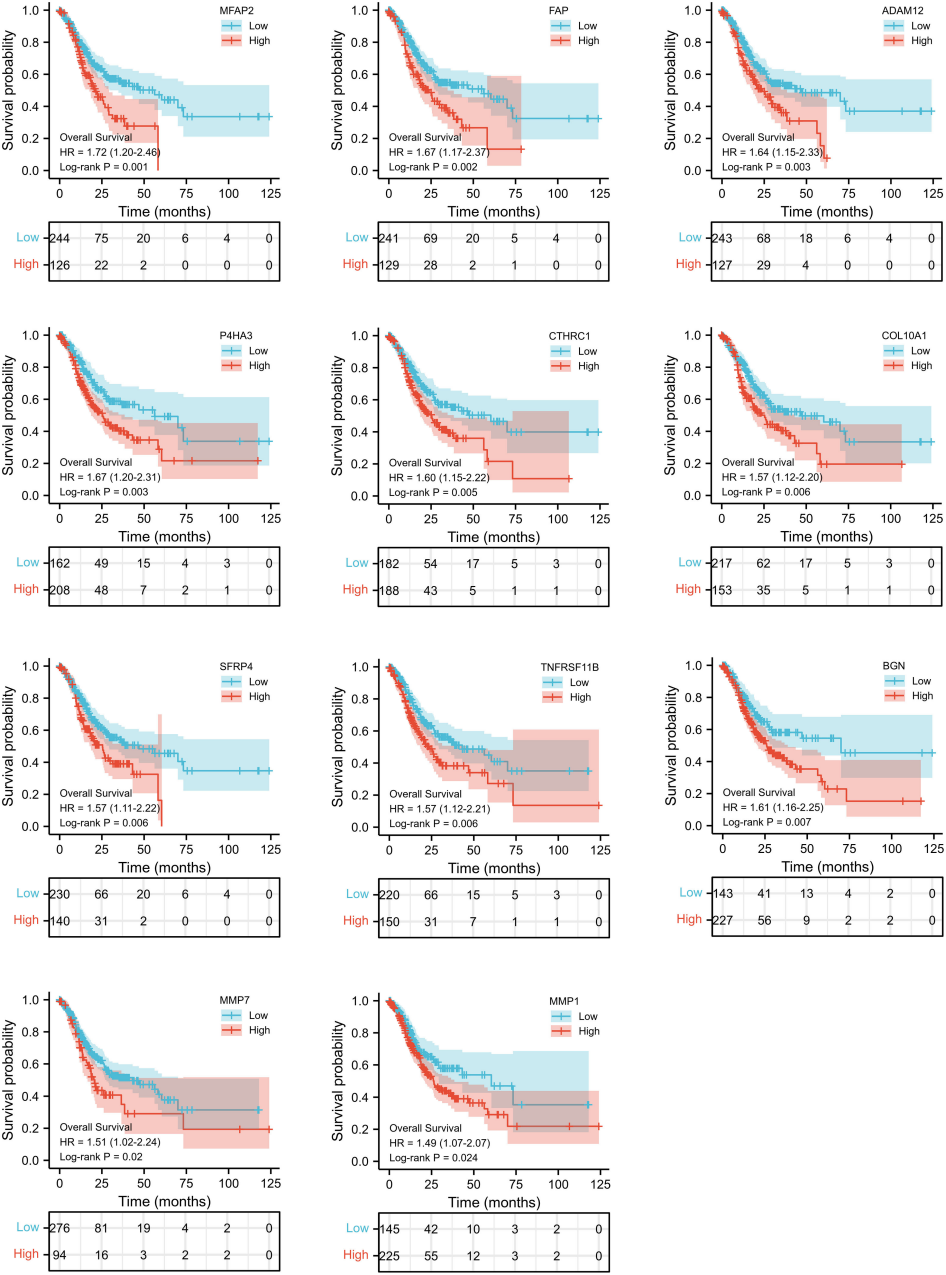
Kaplan–Meier curves of 11 gene with prognostic value.

We selected the top 50 overlapping DEGs (cutoff: *p* < 0.05 and |Log_2_FC| >1.75) as candidate genes. According to log-rank *p* < 0.05 by Kaplan–Meier survival analysis, 11 genes (ADAM2, BGN, COL10A1, MMP1, MMP7, MFAP2, CTHRC1, P4HA3, SFRP4, TNFRSF11B, and FAP) were screened as prognostic-related genes for following research, and the Kaplan–Meier plots were shown in Figure 3.

### Construction and Validation Prognostic Model of STAD

Since the expression value of ADAM2 was zero in half of the samples, it was impossible to group by the median. The results of the univariate and multivariate proportional hazards regression analyses of the 10 prognostic-related genes associated with clinical outcomes are shown in Table 2. Multivariate regression analysis revealed COL10A1, MFAP2, CTHRC1, P4HA3, and FAP as the risk factor, and the risk score formula for OS was as follows:


$$\begin{array}{l} \text{Riskscore }=\;(-0.0013\;\times \text{ COL}10\text{A}1\text{Exp})\;+\;(0.2709\\ \times \;MFAP2Exp)\;+\;(0.1869\;\times \text{ CTHRC}1\text{Exp})\;+\;(0.1649\\ \times \;P4HA3Exp)\;+\;(9\text{e }-\;04\;\times \text{ FAPExp}) \end{array}$$

**Table 2:** Univariate and multivariate Cox regression analyses.

**Characteristics**	**Total (*N*)**	**Univariate analysis**		**Multivariate analysis**	
		**Hazard ratio (95% CI)**	***p-*value**	**Hazard ratio (95% CI)**	***p-*value**
COL10A1 (high vs. low)	370	1.434 (1.030–1.996)	**0.033**	0.999 (0.595–1.676)	0.996
MFAP2 (high vs. low)	370	1.593 (1.140–2.226)	**0.006**	1.311 (0.851–2.021)	0.220
CTHRC1 (high vs. low)	370	1.559 (1.118–2.174)	**0.009**	1.206 (0.708–2.052)	0.491
P4HA3 (high vs. low)	370	1.511 (1.084–2.107)	**0.015**	1.179 (0.740–1.878)	0.488
FAP (high vs. low)	370	1.415 (1.017–1.970)	**0.039**	1.001 (0.598–1.676)	0.997
BGN (high vs. low)	370	1.326 (0.954–1.844)	0.094		
MMP1 (high vs. low)	370	1.187 (0.855–1.648)	0.305		
MMP7 (high vs. low)	370	1.084 (0.781–1.504)	0.631		
SFRP4 (high vs. low)	370	1.267 (0.913–1.758)	0.157		
TNFRSF11B (high vs. low)	370	1.341 (0.966–1.862)	0.080		

**Figure 4:**
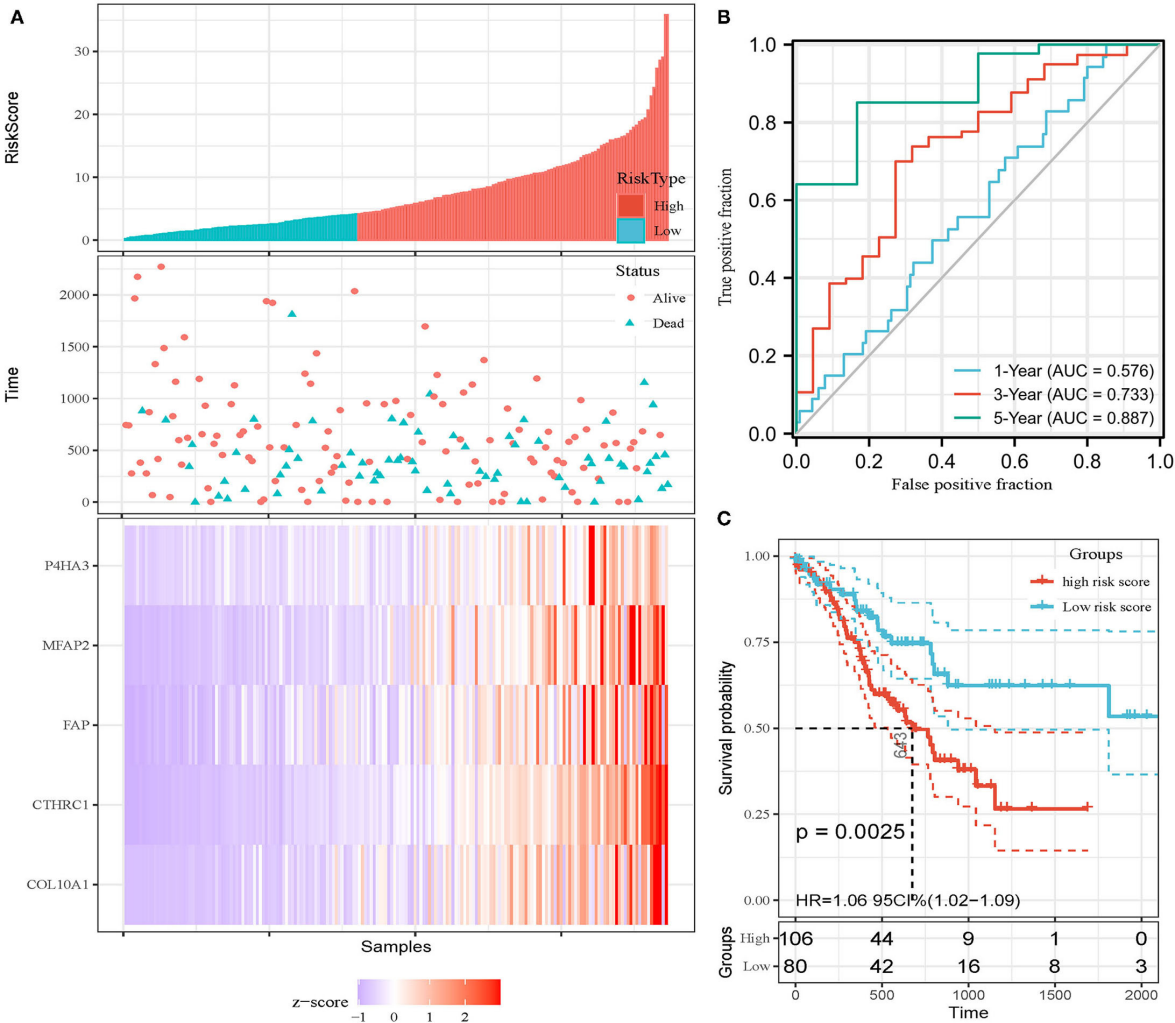
Development of prognostic model in the training set. **(A)** Expression heatmap of five core genes. **(B)** ROC curves for survival risk predicted by risk score for 1-, 3-, and 5-year follow-ups. **(C)** Survival curves of high- and low-risk groups separated by risk score.

**Figure 5:**
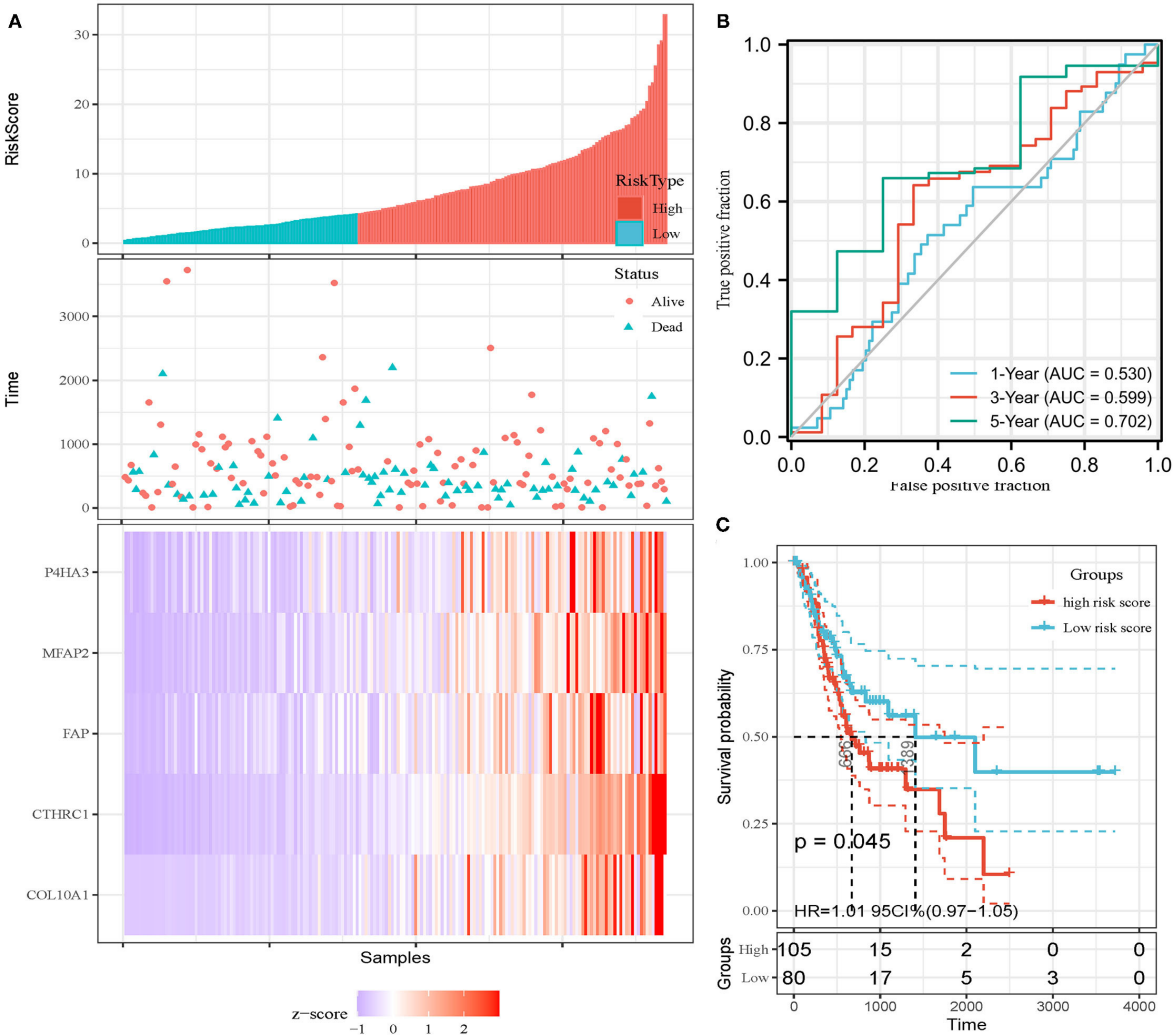
Invalidation of prognostic model in the invalidation set. **(A)** Expression heatmap of five core genes. **(B)** ROC curves for survival risk predicted by risk score for 1-, 3-, and 5-year follow-ups. **(C)** Survival curves of high- and low-risk groups separated by risk score.

In addition, the patients of TCGA-STAD dataset were divided into training set and validation set. Training set consisted of 186 STAD cases whereas validation set consisted of 185 STAD cases. Patients with STAD were divided into high- and low-risk subgroups according to the median value of risk score (cutoff = 14.9). In the training set, the survival analysis showed that the OS rates in the high-risk group were significantly lower than those in the low-risk group (*p* = 0.0025, Figure 4C). The time-dependent ROC curves offered a survival prediction that the AUCs were 0.576 (1-year OS), 0.733 (3-year OS), and 0.887 (5-year OS). Result showed that the risk model had a good ability to predict long-term prognosis of STAD (Figure 4B). The heatmap showed that the expression levels of five core genes were higher in patients with STAD with high-risk scores than those with low-risk scores (Figures 4A, 5A). Meanwhile, data in the validation set showed the similar results: OS rates in the high-risk group were significantly lower than those in the low-risk group (*p* = 0.045, Figure 5C); the time-dependent ROC curves (Figure 5B) predicted that the AUCs were 0.530 (1-year OS), 0.599 (3-year OS), and 0.702 (5-year OS). Moreover, using multivariate Cox regression analysis, the prognostic model was identified as an independent predictor for patients with STAD (*p* = 0.008, Table 3).

**Table 3:** Univariate and multivariate Cox regression analyses for risk score of patients with STAD.

**Characteristics**	**Total (*N*)**	**Univariate analysis**		**Multivariate analysis**	
		**Hazard ratio (95% CI)**	***P* value**	**Hazard ratio (95% CI)**	***P* value**
**Age**	370				
≥65	213	Reference			
≤ 65	157	0.607 (0.430–0.856)	**0.004**	0.538 (0.369–0.785)	**0.001**
**Gender**	370				
Male	237	Reference			
Female	133	0.789 (0.554–1.123)	0.188		
**T stage**	362				
T1&T2	96	Reference			
T3&T4	266	1.719 (1.131–2.612)	**0.011**	1.410 (0.894–2.226)	0.140
**M stage**	352				
M0	327	Reference			
M1	25	2.254 (1.295–3.924)	**0.004**	2.489 (1.337–4.634)	**0.004**
***N*** **stage**	352				
N0&N1	204	Reference			
N2&N3	148	1.650 (1.182–2.302)	**0.003**	1.517 (1.060–2.172)	**0.023**
**Histologic grade**	361				
G1&G2	144	Reference			
G3	217	1.353 (0.957–1.914)	0.087	1.399 (0.956–2.048)	0.084
**Risk score**	370	1.036 (1.012–1.062)	**0.004**	1.037 (1.010–1.065)	**0.008**

### PPI Network Construction and Functional Enrichment Analysis

**Figure 6:**
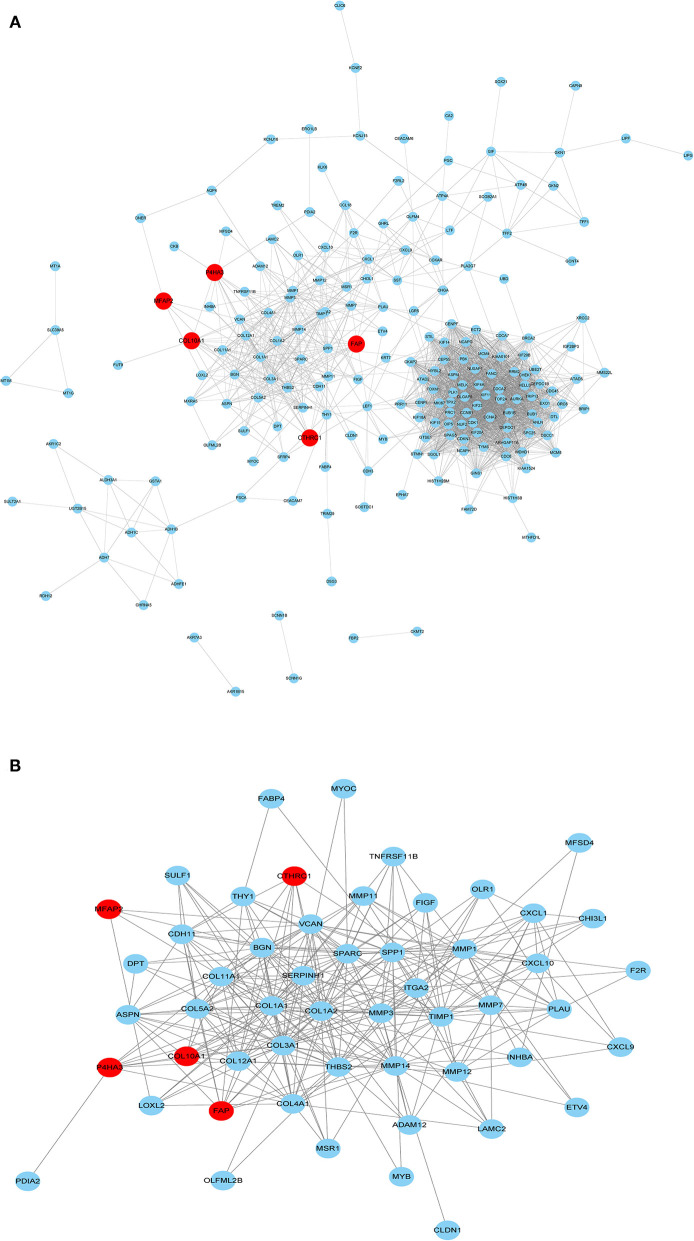
PPI network construction. **(A)** PPI for the five core genes in 229 overlapping DEGs. **(B)** Important modules including the five core genes in the PPI network.

The PPI network of the five core genes was shown in Figure 6A. The topological properties of the PPI network for each gene were shown in Additional File 6. Highly interconnected subcluster of the five core genes was shown in Figure 6B, and the subcluster consisted of 53 nodes and 305 edges (score = 11.731), which represented relatively stable of protein in the network.

**Figure 7:**
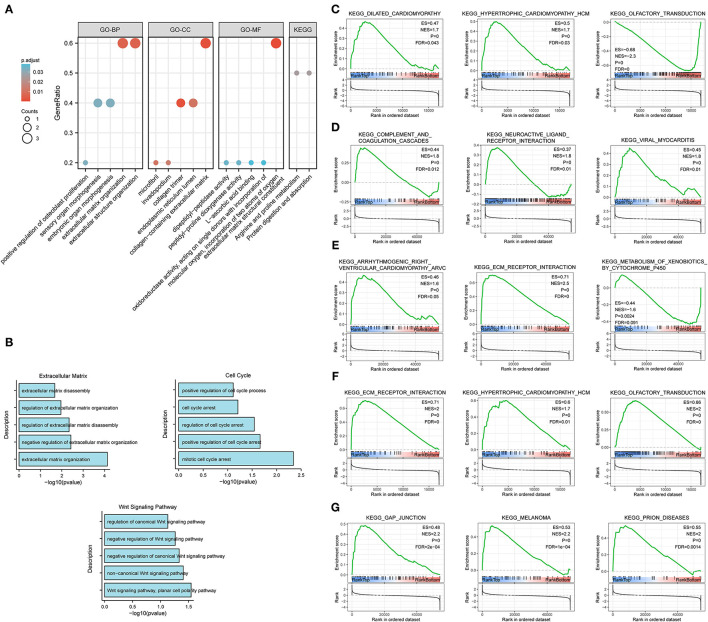
Enrichment analysis for the five core genes. **(A)** GO and KEGG enrichment analysis. **(B)** Biological process enrichment analysis. **(C–G)** GSEA for COL10A1 **(C)**, CTHRC1 **(D)**, MFAP2 **(E)**, P4HA3 **(F)**, and FAP **(G)**.

The results of GO enrichment analysis (Figure 7A) showed that the five core genes significantly focused on extracellular matrix organization, extracellular structure organization (biological process, BP); extracellular matrix structural constituent, dipeptidyl-peptidase activity (molecular function, MF); and collagen-containing extracellular matrix, collagen trimer (cell components, CC). Meanwhile, we found that in terms of biological processes, the genes were mainly focused on extracellular matrix, cell cycle, and Wnt signaling pathways. According to the *p*-value, the top five items from the three categories were selected to plot a histogram (Figure 7B). KEGG enrichment analysis (Figure 7A) indicated that prognostic genes were significantly enriched with arginine and proline metabolism and protein digestion and absorption, etc.

**Figure 8:**
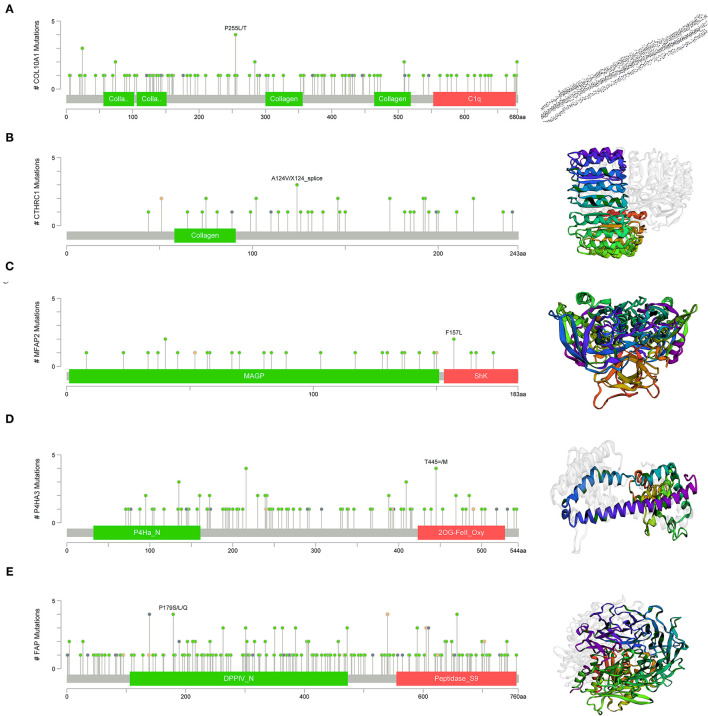
Structural and functional in of the five core genes. **(A)** COL10A1, **(B)** CTHRC1, **(C)** MFAP2, **(D)** P4HA3, and **(E)** FAP.

Gene set enrichment analysis was applied to determine their related signaling pathways (Figures 7C–G). COL10A1 was significantly enriched with olfactory transduction and nitrogen metabolism pathways, *etc*. CTHRC1 was significantly enriched in olfactory transduction and metabolism of xenobiotics by cytochrome p450 pathways, etc. MFAP2 was significantly enriched with olfactory transduction and fatty acid metabolism pathways, etc. P4HA3 was significantly enriched with ribosome and nitrogen metabolism pathways, etc. FAP was significantly enriched in nitrogen metabolism and metabolism of xenobiotics by cytochrome p450 pathways, etc. The mutation site and structure of the five core genes were shown in Figure 8.

### Prognostic Gene Expression Investigation in STAD and Nomogram Construction

**Figure 9:**
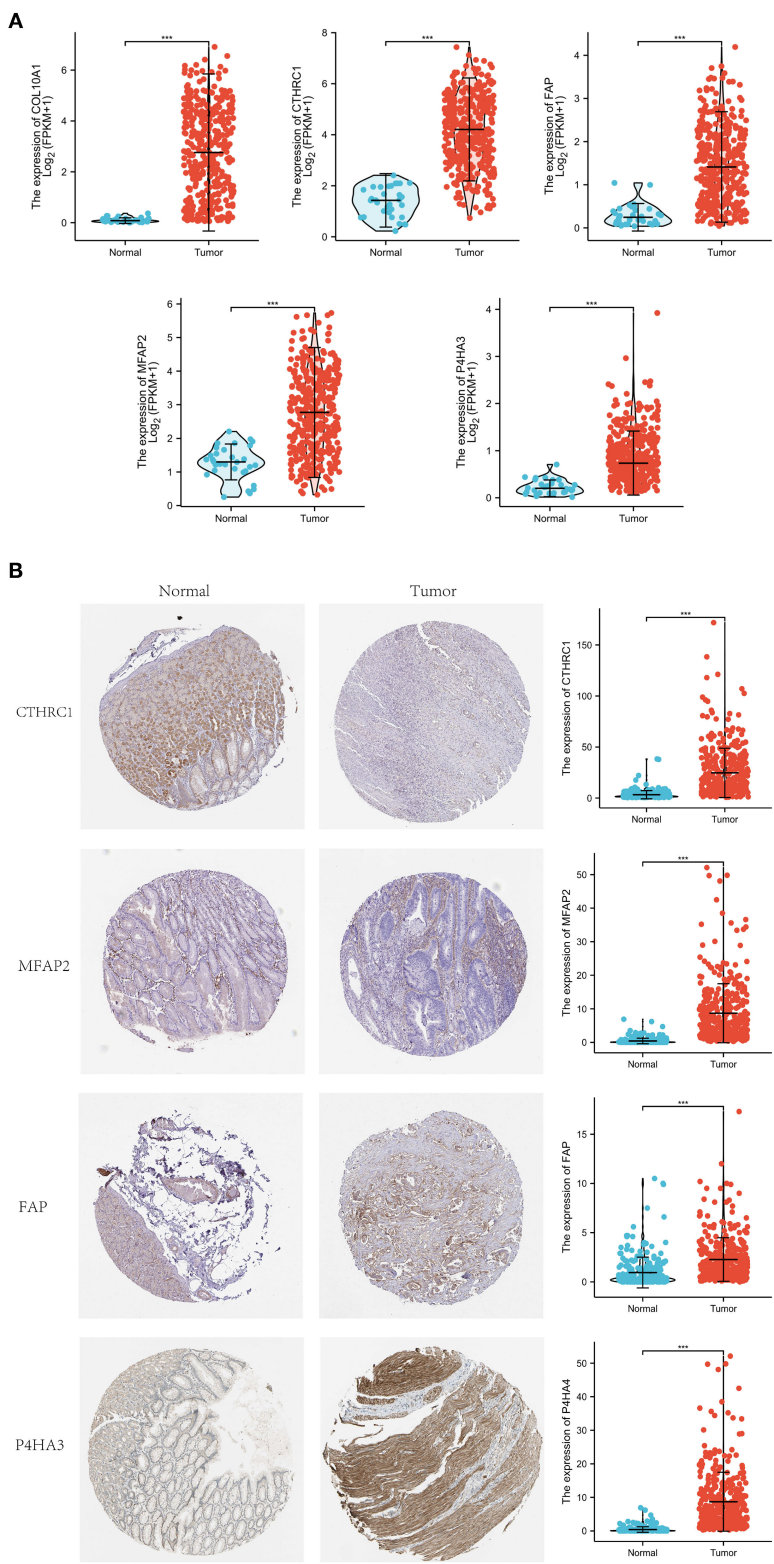
Expression investigation of five core genes. **(A)** Differential expression of the five core genes between normal and STAD-related tissues. **(B)** Immunohistochemistry staining and their mRNA expression in normal and STAD-related tissues based on The Human Protein Atlas. ^***^p < 0.001.

**Figure 10:**
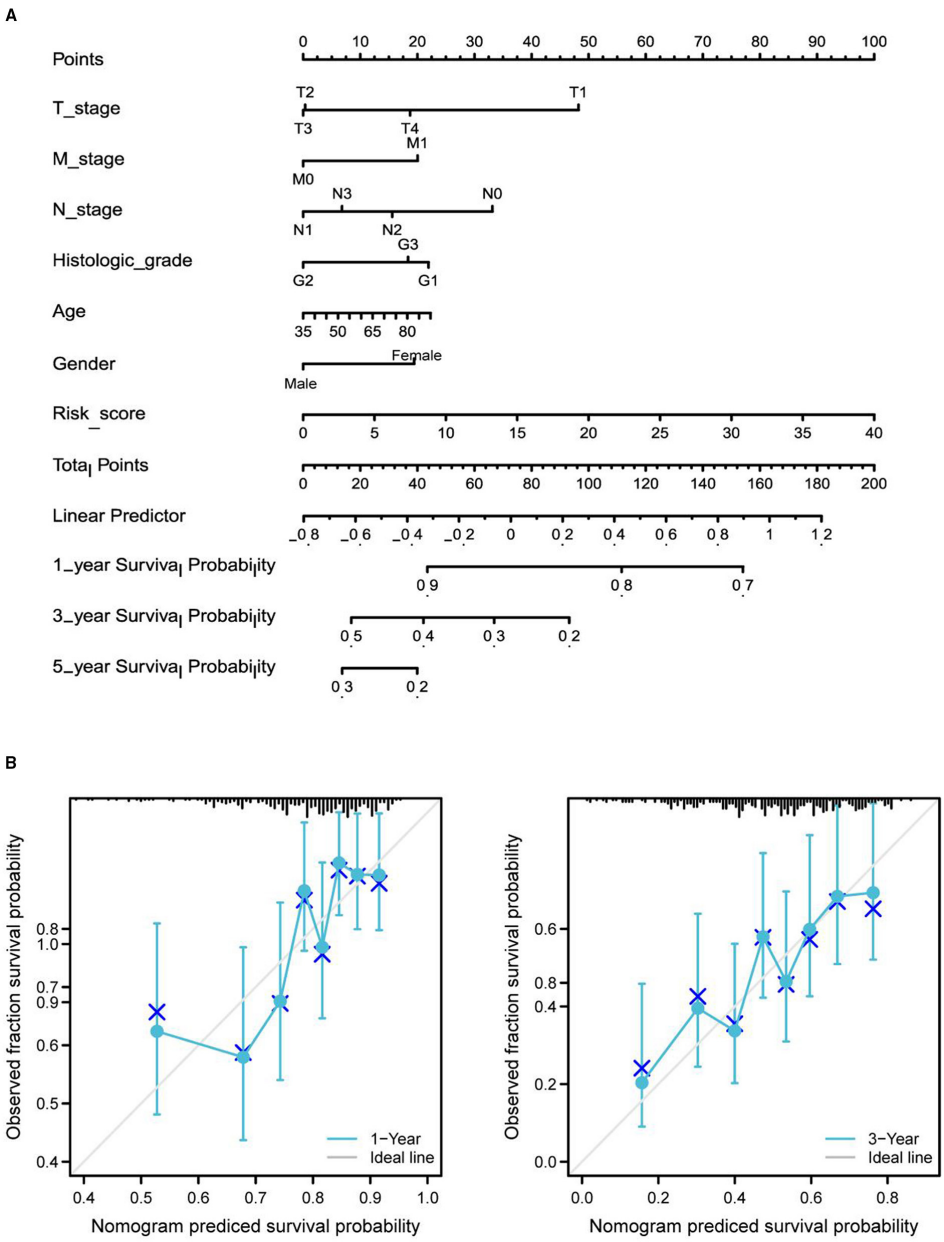
Nomogram predicted the 1-, 3-, and 5-year survival rates of patients with STAD. **(A)** Nomogram predicting the 1-, 3-, and 5-year OS rates of patients with STAD. **(B)** Calibration plots for the 1- and 3-year OS nomogram.

Differential expression of the prognostic genes between normal and STAD-related tissues was verified. Results demonstrated that COL10A1, MFAP2, CTHRC1, P4HA3, and FAP were significantly upregulated in STAD-related tissues compared with normal tissues (Figure 9A). Additionally, immunohistochemistry staining of five core genes in STAD and normal tissues was acquired from the Human Protein Atlas database, which showed that differential expression of protein was consistent with gene expression (Figure 9B). However, the immunohistochemical images of COL10A1 were not found. Then, a nomogram (Figure 10A, including TNM stage, age, gender, histologic grade, and risk score) was created to predict the survival rate of patients with STAD at 1, 3, and 5 years. It was found that high total points predicted low 1-, 3-, and 5-year survival rates; however, a low total points did the opposite. The nomogram calibration plots (Figure 10B) indicate that the nomogram was well-calibrated, with mean predicted probabilities for 1- and 3-year OS close to observed probabilities.

## Discussion

The genetic background of STAD is complicated. Mining genes related to the prognosis of STAD from the genetic and molecular level is of great significance for the treatment and prognosis prediction of STAD. Bioinformatics analysis based on large databases has pointed out the direction for tumor research. In this study, we downloaded gene expression profiling and clinical data from the TCGA and GEO databases, identified DEGs, screened the prognostic-related genes, and then constructed a prognostic model based on five core genes (COL10A1, MFAP2, CTHRC1, P4HA3, and FAP).

COL10A1 is a member of the collagen family involved in tissue architecture and acts as a barrier to the migration of epithelial cells under normal conditions (14). Necula et al. (14) identified a significant increase in COL10A1 plasma level in patients with STAD and concluded that COL10A1 shows an elevated expression from the beginning of carcinogenesis, in the early stages, and its increased level remains elevated during gastric cancer progression. Aktas et al. also found that COL10A1 is abnormally upregulated in gastric cancer and its high expression can be used as a diagnostic and/or prognostic biomarker (15). It has been reported that MFAP2 is upregulated in STAD, negatively correlated with OS, and can be used as a prognostic biomarker of STAD (16, 17), which is consistent with our results. Further, Yao et al. revealed that MFAP2 is overexpressed in gastric cancer and promotes motility *via* the MFAP2/integrin α5β1/FAK/ERK pathway (18). CTHRC1 is a cancer-related gene that can promote cancer recurrence or metastasis *via* diverse signaling pathways, including TGF-β, MEK/ERK, and PKC-δ/ERK (19). Ding et al. (20) found that CTHRC1 promoted STAD metastasis through HIF-1α/CXCR4 signaling pathway, which can be used as a biomarker for STAD, and is consistent with our results. Moreover, CTHRC1 was demonstrated that overexpressed in hepatocellular carcinoma tissues significantly correlating with poor survival rate, which can be used as a prognostic marker for liver cancer (21). Consistent with the results of this study, P4HA3 has been repeatedly reported to be overexpressed in STAD and is related to the poor prognosis of STAD (22). Song et al. found that P4HA3 can be apparently activated by Slug in STAD tissues, of which imbalance and metastasis were related to poor survival rates (23). FAP is a fibroblast activating protein, which has found to be involved in the growth and formation of a variety of cancers. Research revealed that FAP promoted the growth of intrahepatic cholangiocarcinoma through the recruitment of myeloid derived suppression cells (24). Additionally, the high expression of FAP in colorectal cancer is related to angiogenesis and immune regulation (25).

In this study, we established a polygene risk factors model for predicting prognostic of STAD, which is more rational than single-risk factor. However, there are potential limitations to our analysis. First, this study has limitations inherent to a bioinformatics analysis. The construction of prognostic model is based on the TCGA and GEO database analysis and lacks clinical or cellular or animal functional experimental verification. Second, due to some patients with incomplete details are excluded, there may be selection bias in this study.

## Conclusion

We developed a prognostic model for patients with STAD based on COL10A1, MFAP2, CTHRC1, P4HA3, and FAP, and a nomogram to predict the survival rate of patients with STAD at 1, 3, and 5 years. The evidence from this study comes from bioinformatics, as with other studies of a similar nature. It is still necessary to conduct further experiments to verify these findings.

## Data Availability Statement

The datasets presented in this study can be found in online repositories. The names of the repository/repositories and accession number(s) can be found in the article/Supplementary Material.

## Ethics Statement

Ethical review and approval was not required for the study on human participants in accordance with the local legislation and institutional requirements. Written informed consent for participation was not required for this study in accordance with the national legislation and the institutional requirements.

## Author Contributions

YW designed the study. TW and WW analyzed the data and wrote the original manuscript. HL and JZ collected the data. XZ contributed to the manuscript revision. All authors have read and approved the final manuscript.

## Funding

This study was supported by grants from the Zhejiang Provincial National Science Foundation (LY21H270013 and LGF21H310004), China Postdoctoral Science Foundation (2020M671818), Zhejiang Medical and Health Science and Technology Plan (WKJ-ZJ-2136 and 2019RC068), and Hangzhou Medical and Health Science and Technology Plan (2016ZD01, OO20190610, and A20200174).

## Conflict of Interest

The authors declare that the research was conducted in the absence of any commercial or financial relationships that could be construed as a potential conflict of interest.

## Publisher's Note

All claims expressed in this article are solely those of the authors and do not necessarily represent those of their affiliated organizations, or those of the publisher, the editors and the reviewers. Any product that may be evaluated in this article, or claim that may be made by its manufacturer, is not guaranteed or endorsed by the publisher.

## Abbreviations

STAD: stomach adenocarcinoma
GEO: Gene Expression Omnibus
TCGA: the Cancer Genome Atlas
DEGs: differentially expressed genes
TCGA-STAD: The Cancer Genome Atlas- Stomach Adenocarcinoma
PPI: protein–protein interaction
OS: overall survival
ROC: receiver operating characteristic
AUCs: areas under ROC curves
STRING: Search Tool for the Retrieval of Interacting Genes database
GO: gene ontology
KEGG: Kyoto Encyclopedia of Genes and Genomes
BP: biological process
MF: molecular function
CC: cell components
GSEA: gene set enrichment analysis
CRC: colorectal cancer
FDR: false discovery rate.
